# Deregulated Expression of Circular RNAs Is Associated with Immune Evasion and Leukemia Relapse after Allogeneic Hematopoietic Stem Cell Transplantation

**DOI:** 10.3390/genes13111986

**Published:** 2022-10-31

**Authors:** Fei Zhao, Xiaoyu Zhang, Xiaolei Pei, Donglin Yang, Mingzhe Han

**Affiliations:** State Key Laboratory of Experimental Hematology, National Clinical Research Center for Blood Diseases, Haihe Laboratory of Cell Ecosystem, Institute of Hematology & Blood Diseases Hospital, Chinese Academy of Medical Sciences & Peking Union Medical College, Tianjin 300020, China

**Keywords:** allogeneic hematopoietic stem cell transplantation, relapse, circRNA, immune evasion, acute myeloid leukemia

## Abstract

Background: Circular RNAs (circRNAs) are a novel class of epigenetic regulators that participate in leukemogenesis. However, their roles in leukemia relapse after transplantation remain unclear. Methods: We defined the circRNAs profile of the bone-marrow-enriched CD34^+^ cells from ten acute myeloid leukemia (AML) patients after transplantation (five relapse [RE] and five continuous complete remission [CR]) and four healthy controls (HCs) by RNA-seq. Differentially expressed circRNAs were validated using real-time quantitative polymerase chain reaction (RT-qPCR) in an independent cohort of six AML patients with pairwise samples at diagnosis and at relapse and six controls. Results: The bioinformatics analysis revealed a distinct circRNAs profile in relapse patients compared with controls (CR or HCs), while there was no significant difference between CR and HCs. Functional enrichment analysis demonstrated that mRNAs co-expressed with identified circRNAs were primarily involved in immune-related pathways, including the T cell receptor signaling pathway and lymphocyte differentiation. Moreover, we performed a protein–protein interaction network based on the immune-related genes and annotated 20 hub genes. The abnormal expression of hub genes was responsible for impairing T cell co-stimulation and activation, thus contributing to the immune escape of relapse blasts. We further constructed competing endogenous RNAs (ceRNA) regulatory networks based on immune-related genes and identified 10 key circRNAs that are associated with immune evasion. Six candidate circRNAs and their associated miRNA/mRNAs in the ceRNA network were randomly selected to be validated in another set by RT-qPCR. Conclusions: CircRNAs dysregulation may be involved in the immune evasion of relapse blasts and is associated with AML relapse. Our results identify several promising biomarkers and might provide novel insights into the biology of AML relapse post-transplantation.

## 1. Introduction

Allogeneic hematopoietic stem cell transplantation (allo-HSCT) is a curative treatment approach for hematological malignancies such as acute myeloid leukemia (AML) [1]. However, relapse remains a major challenge, and the clinical outcomes of relapsed patients have not significantly improved over the past decades [2]. The molecular mechanisms associated with post-transplantation relapse remain elusive, which has restricted more powerful therapeutic armamentarium against leukemia relapse.

In recent years, many researchers have studied the nature of AML relapse. There is growing evidence that the escape of control of allogenic immune responses may contribute to relapse after transplantation, including the downregulation of major histocompatibility complex (MHC) class II genes, the genomic loss of human leukocyte antigen (HLA) and the enforcement of inhibitory checkpoints [3,4]. Moreover, previous studies have demonstrated that relapse after transplantation is in some cases associated with transcriptional changes, not the acquisition of previously unknown leukemia-specific mutations or structural variations, indicating potential epigenetic origins [5,6].

As an important epigenetic component, the discovery of noncoding RNAs (ncRNAs) has opened up broad prospects for cancer diagnosis, prognosis and treatment [7]. NcRNAs regulate gene expression by regulating transcription, post-transcriptional modification and translation, including microRNA (miRNA), long non-coding RNA (lncRNA) and circular RNA (circRNA) [8]. CircRNAs are a class of abundant, single-stranded ncRNA that result from the ligation of a downstream splice donor to an upstream splice acceptor which have been extensively studied in recent years [9,10]. In contrast with linear RNAs, circRNAs are characterized by exceptionally high stability, tissue-specific expression and evolutionary conservation. CircRNAs can regulate downstream gene expression by directly or indirectly altering transcription and translation through several mechanisms, including sponging miRNAs as competing endogenous RNAs (ceRNAs), serving as templates of translation, sequestering RNA-binding proteins (RBPs) and regulating RBPs interactions [11]. Accumulating evidence indicates that the dysregulation of circRNAs is implicated in a variety of physiologic and pathologic processes, including immune regulation and leukemogenesis [12,13,14]. Recent studies have supported that the dysregulation of immune-related pathways may be mainly responsible for the post-transplant relapse [4,15]. These findings prompted us to interrogate whether the expression of circRNA is altered during AML relapse after transplantation.

In the present study, we provide novel data on the global expression pattern of circRNAs during AML relapse after allo-HSCT. We identified key genes regulated by differentially expressed circRNAs compared with controls. Furthermore, we also constructed the ceRNAs regulatory network to evaluate the crucial circRNAs in buffering the regulatory activity of miRNAs and consequently their influence on the transcription of annotated protein genes. Our results show that the dysregulation of circRNAs may play an important role in the allogeneic immune responses and provide new insight into the underlying mechanisms of leukemia relapse after transplantation.

## 2. Materials and Methods

### 2.1. Patients and Samples

Given the important role of the post-transplantation immune environment in driving altered gene expression, we enrolled a discovery cohort (n = 14) and a validation cohort (n = 12) on the basis of sample availability. The discovery cohort consisted of 14 participants, including 5 AML patients with relapse after allo-HSCT (RE group), 5 patients with continuous complete remission for more than 2 years after transplantation (CR group) and 4 healthy controls (HC group). To further confirm the roles of key circRNAs identified in the discovery cohort in the AML relapse, we selected another validation cohort (n = 12) of 6 AML patients with pairwise samples at diagnosis and at relapse after allo-HCT and 6 controls (HCs = 3, CR = 3). All patients were diagnosed as AML-M5 according to the French–American–British (FAB) classification and underwent the first allo-HSCT between May 2019 and November 2020 in the Hematopoietic Stem Cell Transplantation Center of Blood Diseases Hospital, Chinese Academy of Medical Sciences. Relapse is morphologically diagnosed as more than 5% blast cells in the bone marrow (BM) or the appearance of extramedullary leukemic lesions. In the exploratory stage, we performed RNA-seq of BM samples from discovery cohort. Subsequently, RNA samples were isolated from an independent cohort of 12 participants (n = 6 per group) and were subjected to quantitative analysis for further validation. The clinical characteristics of these participants are shown in Table 1.

The BM aspirations from all of the participants were harvested after informed consent approved by the Ethical Committee of Institute of Hematology & Blood Diseases Hospital. In detail, the BM samples were collected at the median of 160 days (interquartile range [IQR]: 127 to 243) and 124 days (IQR: 108 to 180) after transplantation from patients in the CR and RE group, processed to mononuclear cells with density centrifugation and cryopreserved in liquid nitrogen for further analyses. All relapse samples showed high expressions of the CD34 marker, and the AML blasts counts were approximately 80% in the clinical data. The HLA loss was negative in all of the relapse samples.

### 2.2. Isolation of CD34^+^ Cells and Flow Cytometry

The cryopreserved BM samples were thawed and the CD34^+^ cells were enriched by positive selection using CD34 microbeads and a magnetic cell-sorting system (Miltenyi). Briefly, the BM cell were suspended in 100 μL of PBS/0.5% BSA/2 mM EDTA solution and were filtered through a 30 µm nylon mesh to remove cell clumps. Human FcR blocking reagent and anti-CD34 microbeads were added and then incubated at 4 °C for 30 min. Subsequently, cells were washed and resuspended in 500 μL PBS solution and were passed through the MS separation column kept in the magnetic field to hold the CD34^+^ cells in the column. Then, the purity of the isolated CD34^+^ cells was evaluated by flow cytometry; the post-sort purity was routinely >95% (Figure S1). In detail, 1 × 10^5^ cells were suspended in 100 μL PBS and were stained with FITC-conjugated anti-CD34 (Biolegend) in the dark for 30 min on ice, and DAPI (1 μg/mL) was added to gated-out dead cells before running the samples. The samples were stained and analyzed on a FACS Canto II flow cytometer, and data was processed with FlowJo version 9 or 10 (Tree Star).

### 2.3. Whole Transcriptome Sequencing (WTS)

The total RNA from purified the CD34^+^ cells (2–3 × 10^6^) was extracted using TRIzol reagent (Thermo Fisher) according to the manufacturer’s protocol. The quality and quantity of total RNA was measured using Nanodrop 2000 (Thermo Fisher Scientific, Waltham, MA, USA). The RNA libraries were prepared with the TruSeq Stranded Total RNA Ribo-Zero Gold kit. An Agilent 2100 Bioanalyzer (Agilent Technologies, Palo Alto, CA, USA) was used to test the RNA integrity, and samples with an RNA integrity number (RIN) ≥7 were subjected to subsequent analysis. Small RNA (miRNA) and RNA (circRNA, lncRNA and mRNA) library construction was conducted and sequenced on the Illumina Hiseq2500 platform at an average sequencing depth of 140 million reads per sample. CircRNAs were detected and quantified by CirComPara v0.6 using 9 backsplice detection methods and default parameters.

### 2.4. Real-Time Quantitative Polymerase Chain Reaction (RT-qPCR)

In the validation cohort, 6 AML patients with pairwise samples at diagnosis and at relapse after allo-HCT and 6 controls (HCs = 3, CR = 3) were enrolled for quantitative analysis. Briefly, total RNA was extracted from primary BM CD34^+^ cells with TRIzol reagent and then first-strand cDNA was synthesized with a PrimeScript RT reagent Kit with gDNA Eraser (TAKARA, Otsu, Japan) according to the manufacturer’s instructions. RT–qPCR for the miRNAs was performed on cDNA generated from 0.5 μg total RNA according to the manufacturer’s protocol (Ribobio). U6 was used for miRNAs template normalization and GAPDH was used for circRNAs and coding genes template normalization, respectively. Quantitative validation was performed using a Fast SYBR Green Master Mix (Roche, Basel, Switzerland) on a QuantStudio 5 real-time PCR detector (ThermoFisher, Waltham, MA, USA) for DE circRNAs/miRNAs/mRNAs identified in the ceRNAs network. All primers used in this study are listed in Supplemental Table S1.

### 2.5. Identification of Differentially Expressed Genes

Purified CD34^+^ blasts or hematopoietic stem progenitor cells (HSPCs) from all patients and HCs were analyzed after quality control analysis. RNA sequencing reads were mapped to the GRCh38.p7 obtained from NCBI. Gene expression was normalized to fragments per kilobase million (FPKM) and analyzed further by log_2_ (FPKM+1) transformation. Differential expression genes were determined by DEseq2 and |log_2_Fold change (FC)| > 1 with a *p* value < 0.05 as the threshold for statistical significance. The absolute value of a Pearson correlation coefficient >0.99 was defined as co-expression between differential expressions of circRNAs and mRNAs (DE circRNAs and DE mRNAs) according to the expression levels.

### 2.6. Functional Annotation and Enrichment Analysis

Gene ontology was conducted to annotate the co-expressed mRNAs with terms under the biological process (BP) (http://www.geneontology.org, accessed on 22 April 2022). The analyses of the signaling pathways with enriched mRNAs were based on the Kyoto Encyclopedia of Genes and Genomes (KEGG) database (https://www.genome.jp/kegg, accessed on 1 May 2022).

### 2.7. CeRNA Regulatory Network Analysis

The target miRNAs of DE circRNAs and co-expressed mRNAs were predicted by the CircNet, TargetScan and miRTarBase databases. Any overlapping of the same miRNAs was considered to be potential circRNA–miRNA–mRNA interactions. The ceRNA regulatory network was visualized using the Cytoscape software.

### 2.8. Statistics

The results are summarized as median ± interquartile range (IQR) where appropriate. The *p* values are two-sided and a value < 0.05 is considered statistically significant. All analyses were performed with Graph Pad Prism version 8.0 (Graph Pad, San Diego, CA, USA).

## 3. Results

### 3.1. Overview of Global circRNAs Profiles in AML Patients with Relapse after Transplantation

**Figure 1:**
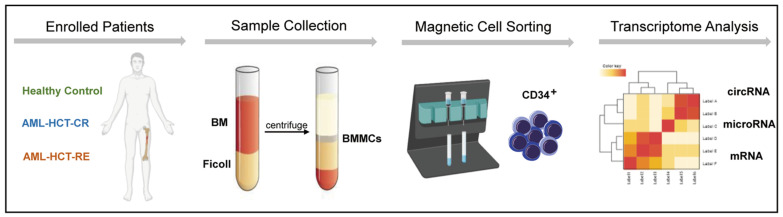
Schematic of RNA-seq analysis of BM-purified CD34^+^ cells from AML patients with relapse (RE), remission (CR) and healthy controls (HC).

To explore the potential effect of circRNAs on post-transplant relapse, we first performed RNA-seq on purified BM CD34^+^ cells from AML patients who received first allo-HSCT (CR and RE, *N* = five per group) and healthy controls (HC, *N* = four) in the discovery set (Figure 1). In general, we identified on average 9880 different circRNAs per sample in the RE group, 9562 circRNAs in the CR group and 9500 circRNAs in the HC group. The principal component analysis indicated distinct circRNAs profiles among the groups (Figure S2).

### 3.2. CircRNAs Expression Specifically Correlates with Post-Transplant Relapse

**Figure 2:**
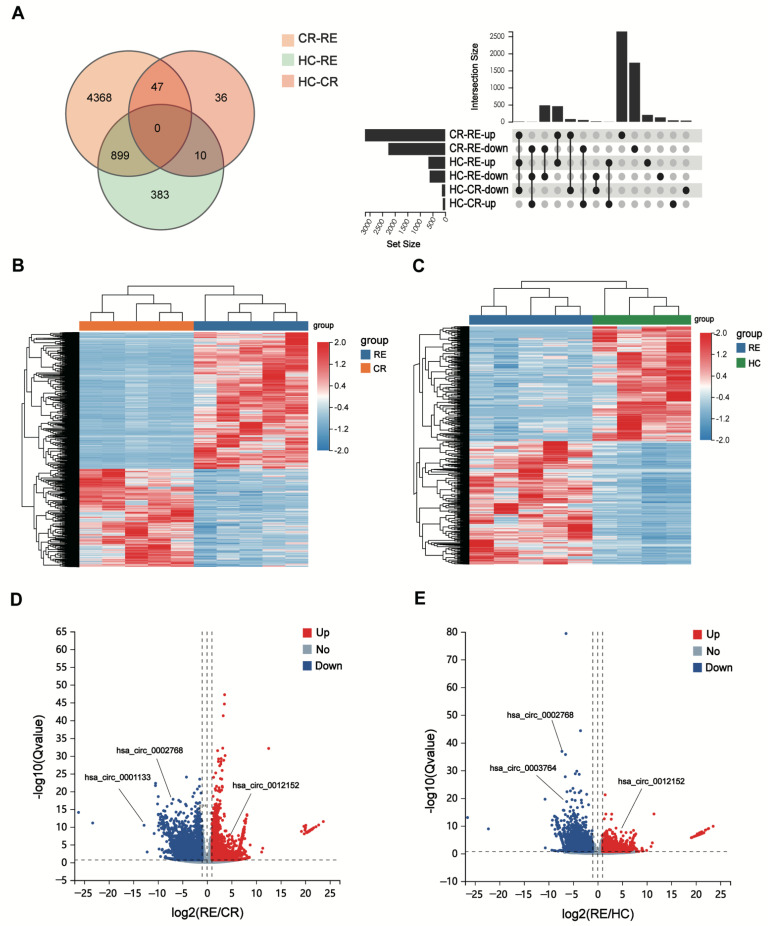
CircRNA expression is altered in AML relapse (RE) compared with controls (CR or HC). (**A**) The Venn diagram (left) and UpSet plot (right) show that numbers of DE circRNAs among the different groups. (**B**,**C**) The heatmaps show the DE circRNAs from unsupervised hierarchical clustering present in AML patients with relapse versus remission (**B**) and relapse versus healthy controls (**C**–**E**). Volcano plot graphs for circRNAs expression levels in patients with relapse versus remission (**D**) and relapse versus healthy controls (**E**) Red indicates an upregulated expression and blue represents a downregulated expression.

To elucidate the unique expression profile of circRNAs from relapse blasts, we conducted statistical analysis following the criteria as mentioned above (|log_2_Fold change (FC)| > 1 and *p* value < 0.05). The circRNAs expression profile in the RE group significantly differed from those in the CR and HC groups (Figure 2A). In contrast, there was not a clear distinction in circRNAs expression between patients in the CR and HC groups (Figure S3). A total of 5314 DE circRNAs (3057 upregulated and 2257 downregulated) were identified between the RE and CR groups (Figure 2B,C). For relapse patients, there were 1292 DE circRNAs (674 upregulated and 618 downregulated) versus controls (Figure 2D,E). In summary, these data demonstrate that striking differences in the expression of circRNAs expression exist in relapse blasts, supporting the important role of circRNAs in post-transplant relapse.

**Figure 3:**
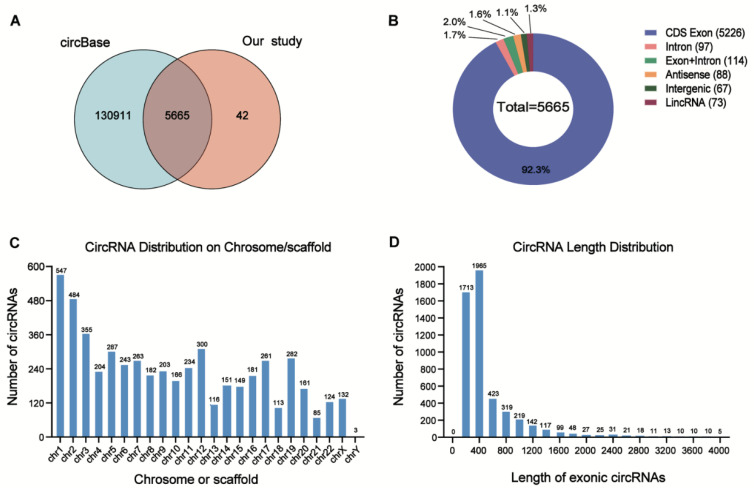
Overview of the DE circRNAs in AML patients with relapse (RE) and controls (CR or HC). (**A**) Overlapping of identified circRNAs between circBase and our study. (**B**) Genomic origin of the circRNAs identified in the relapsed samples and controls. (**C**) The chromosome distributions of exonic circRNAs. (**D**) The length distributions of exonic circRNAs.

Among these DE circRNAs, 5665 circRNAs (99.3%) have been identified in circBase [16], and the others (0.7%) are novel (Figure 3A). The genome distribution of DE circRNAs reveals that the majority of the identified candidates (5226/5665, 92.3%) are transcribed from protein-coding exons (Figure 3B). As is consistent with a previous study [17], the DE circRNAs were widely distributed among chromosomes, and most of the identified circRNAs were located on the chromosomes 1 and 2 (Figure 3C). Moreover, the lengths of most exonic circRNAs were around 200–450 nucleotides (Figure 3D).

### 3.3. Co-Expression Analysis of DE circRNAs and mRNAs and Functional Enrichment

**Figure 4:**
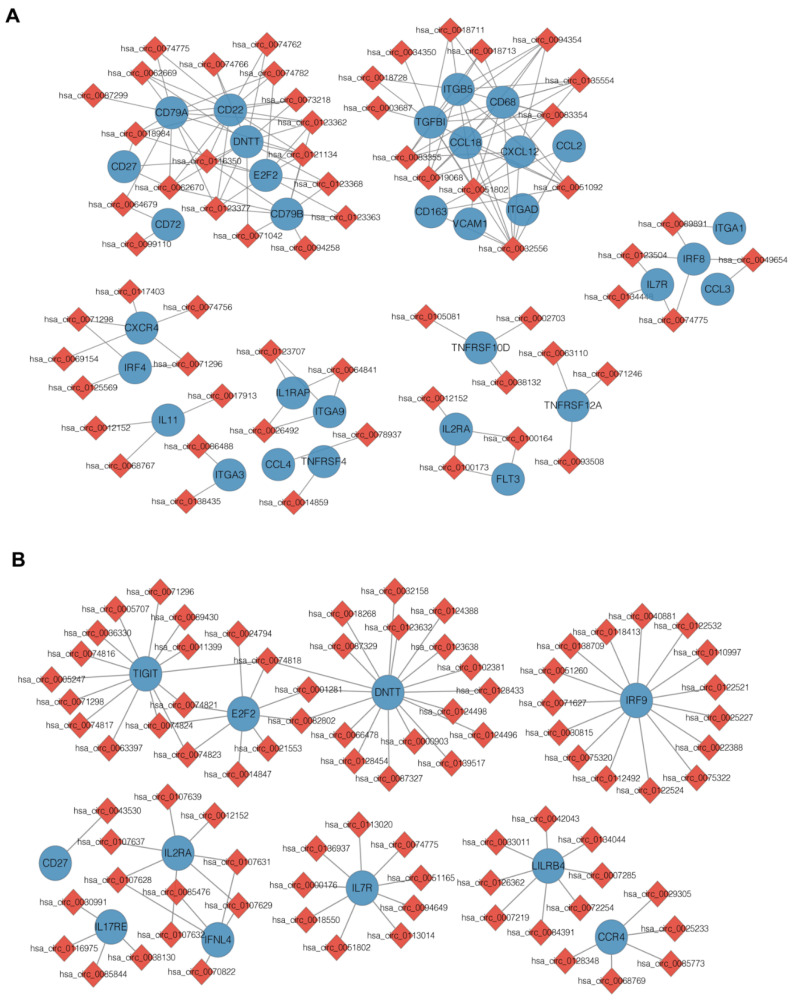
DE circRNAs and mRNAs co-expression networks in the immune-related pathways. (**A**) Co-expression network of the DE circRNAs and DE mRNAs present in the CD34^+^ cells from patients with AML relapse versus remission. (**B**) Co-expression network of the DE circRNAs and DE mRNAs present in the CD34^+^ cells from patients with AML relapse versus healthy controls.

Alterations in circRNA expression can result in an aberrant expression of genes that contribute to myeloid leukemogenesis [8,18]. Thus, we constructed the circRNA–mRNA co-expressed network based on the correlation analysis (|PCC| > 0.99) between DE circRNAs and DE mRNAs in the relapse samples versus the HCs. The co-expression analysis revealed that 311 DE mRNAs (98 upregulated and 213 downregulated) had a strong correlation with 153 DE circRNAs (90 upregulated and 63 downregulated) between the RE and CR groups (Figure 4A and Figure S4). In the RE group, 166 DE mRNAs were co-expressed with 239 DE circRNAs compared with the HCs (Figure 4B and Figure S5).

**Figure 5:**
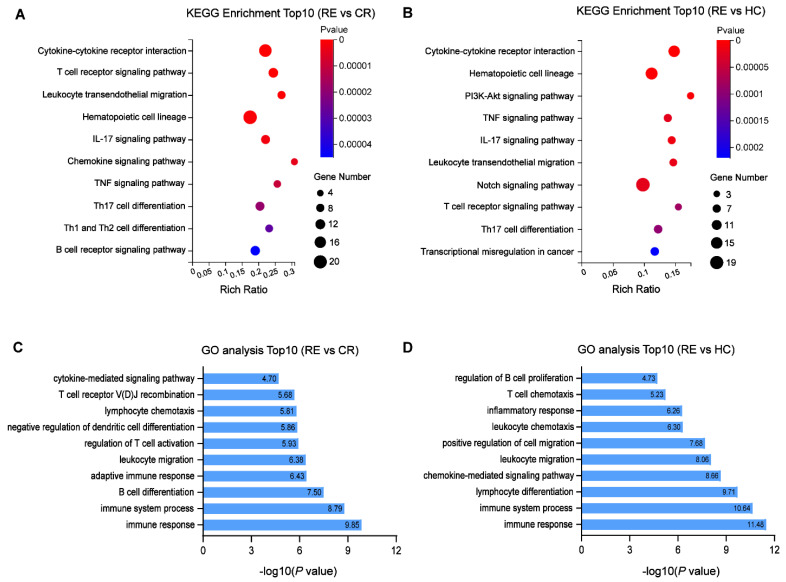
Functional enrichment analysis for the DE mRNAs co-expressed with identified DE circRNAs. (**A**,**B**) Top10 KEGG pathways enriched in AML relapse compared with remission (**A**) or healthy controls. (**B**–**D**) Top10 biological processes of GO terms enriched in AML relapse compared with remission (**C**) or healthy controls (**D**).

To clarify the potential functions of identified DE circRNAs, we conducted GO and KEGG pathway analyses based on the co-expressed genes. Of note, the co-expressed genes in both pairs were enriched in similar pathways. The KEGG analysis revealed the top 10 enriched pathways among these DE mRNAs, including cytokine–cytokine receptor interaction, T cell receptor-signaling pathway and T cell differentiation (Figure 5A,B). In the GO analysis, DE mRNAs related to biological processes that were mainly enriched in the immune response, immune system process, lymphocyte differentiation and leukocyte migration were identified (Figure 5C,D).

### 3.4. CircRNAs Dysregulation Correlates with Immune Evasion during Post-Transplant Relapse

**Figure 6:**
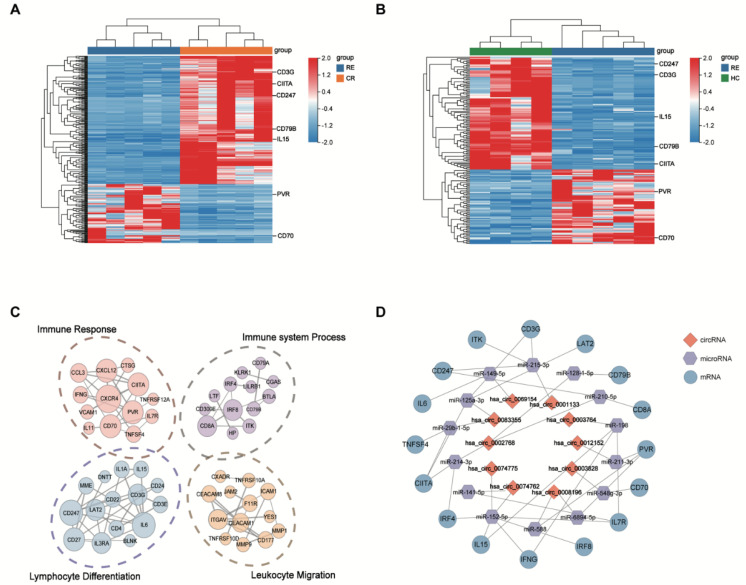
The DE circRNAs identified in relapse blasts regulate immune-related mRNAs. (**A**,**B**) Heatmaps show that immune-related DE mRNAs co-expressed with DE circRNAs from unsupervised hierarchical clustering present in AML patients with relapse versus remission (**A**) and relapse versus healthy controls. (**B**,**C**) Protein–protein interaction analysis shows the significantly altered expression pattern in the immune-related genes regulated by DE circRNAs in relapse blasts. Edges indicate scores based on the STRING database, and node size indicates shared genes. (**D**) The construction of a circRNA–miRNA–mRNA ceRNA network based on the selected hub genes related to immune regulation. The ceRNA regulatory network includes 16 mRNAs, 14 miRNAs and 10 circRNAs. The red square nodes represent circRNAs, the purple hexagonal nodes indicate miRNAs and the blue circular nodes denote mRNAs.

Several previous studies have documented that immune evasion plays a crucial role in mediating relapse after transplantation [15,19]. Consistently, GO analysis has suggested that co-expressed DE mRNAs are primarily involved in immune-related pathways in our study, such as immune response and immune system process. Notably, our results demonstrate downregulation in T cell co-stimulation molecules (HLA class II regulators [CIITA, IRF8, IRF4], the T cell receptor-signaling pathway [CD247, CD3E, CD3G, ITK], activated molecules [LAT2, TNFSF4, IFNG, IL15]) and the up-regulation of inhibitory T cell ligand (PVR) in the RE group, which are known to drive relapsed AML cells to evade from immune control (Figure 6A,B). Our results indicate that DE circRNAs are implicated in promoting the immune-evasion of leukemia cells by regulating the expression of immune-related genes, and thus contribute to post-transplantation relapse. In our subsequent analysis, we focused more attention on these identified DE circRNAs and mRNAs.

To further explore the interaction between immune-related genes, we constructed protein–protein interaction (PPI) networks based on the identified DE mRNAs, which were involved in the immune response, immune system process, lymphocyte differentiation and leukocyte migration (Figure 6C). A total of 20 DE mRNAs predicted by cytoHubba algorithms were identified as hub genes, such as CD247, PVR, IRF8, CD79B, CD70 and TNFSF4 (Table 2).

### 3.5. Construction of the circRNA-miRNA-Immune-Related mRNA Regulatory Network

Accumulating evidence indicates that circRNAs can indirectly regulate mRNA expression by sponging miRNA to relieve the repression of their target genes [20,21] To elucidate the role of circRNA–miRNA–mRNA interactions (ceRNA) during leukemia relapse, we constructed a ceRNA regulatory network depending on immune-related genes and their co-expressed circRNAs. We identified 15 circRNA–miRNA pairs and 27 miRNA–mRNA pairs using the CircNet, TargetScan and miRTarBase databases. The ceRNA network (including 10 circRNAs, 14 miRNAs and 16 mRNAs) was built by integrating with these relationship pairs (Figure 6D). As shown in the ceRNA network, 10 key circRNAs might act as potential ceRNAs that sponged miRNAs to regulate the expression of immune-related genes. In sum, these data indicate that dysregulated ceRNA might be involved in the process of AML relapse after transplantation.

### 3.6. Validation of DE circRNAs by RT-qPCR

**Figure 7:**
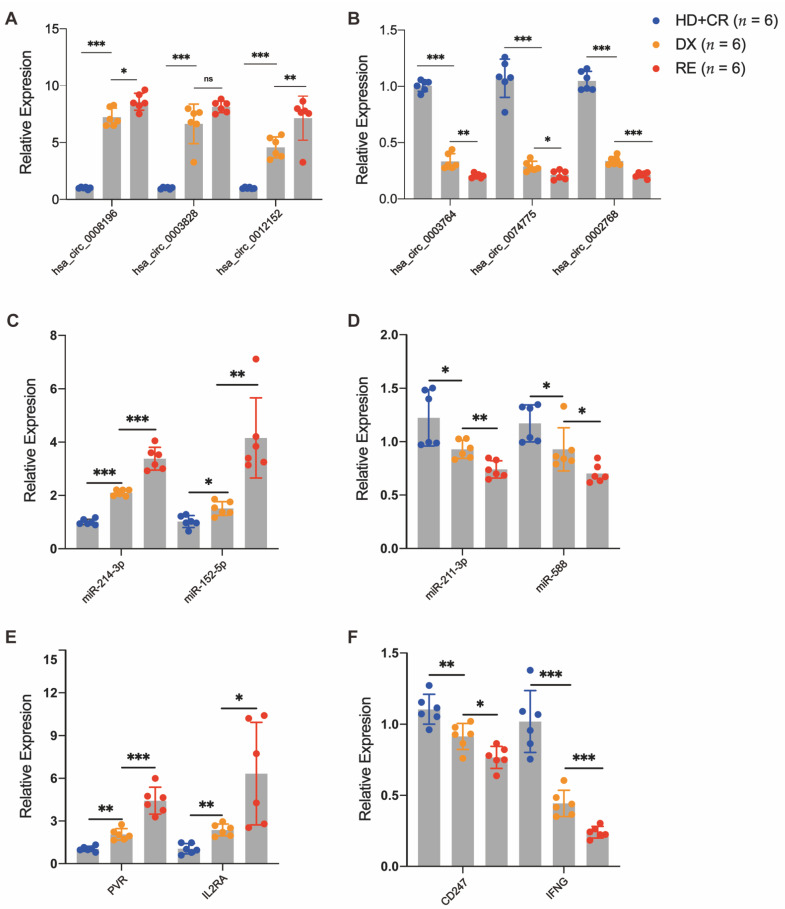
Validation of DE circRNAs in AML patients with pairwise diagnosis (DX) and relapse (RE) and controls (CR or HC)**.** The RT-qPCR detected the relative expression levels of DE circRNAs (**A**,**B**) and their associated DE miRNAs (**C**,**D**) and DE mRNAs (**E**,**F**), which were selected randomly from the ceRNA regulatory network in an independent cohort (*n* = 6 per group). * *p* < 0.05; ** *p* < 0.01; *** *p* < 0.001.

To verify the reliability of the DE circRNAs detected by the transcriptome sequencing, six DE circRNAs (three upregulated and three downregulated) and their associated miRNAs/mRNAs were randomly selected from our ceRNA regulatory network and further verified using RT-qPCR in the validation cohort. We selected six AML with pairwise samples purified at diagnosis and at relapse and six controls. Our results demonstrate that most of the DE circRNAs (5/6, 83.3%) were differentially expressed between pairwise diagnosis and relapse blasts (Figure 7A,B). The results of RT-qPCR in the validation cohort are consistent with those of RNA-seq in the discovery cohort, suggesting that the alteration of DE circRNAs expression provides a clear distinction between AML relapse and controls.

## 4. Discussion

Relapse still represents the major cause of treatment failure, and up to 50% of AML patients finally relapse after allo-HSCT [2,22]. The dismal success of existing therapies underscores the importance of determining the underlying mechanisms of relapse and exploring novel treatment protocols. In the present report, we disclose the expression profile of circRNAs in BM CD34^+^ cells from AML patients with post-transplant relapse and controls, first providing the crucial circRNAs that are implicated in post-transplant relapse. Specifically, we revealed a distinct pattern of circRNA expression in relapse blasts compared with healthy or HSPCs in remission using RNA-seq. We subsequently identified the co-expressed mRNAs to investigate the potential regulatory roles of DE circRNAs. The CeRNA regulatory network analysis showed that hub genes regulated by DE circRNAs are primarily enriched in immune-related pathways, thus indicating their potential roles in immune evasion during relapse after transplantation. These results were validated and confirmed by RT-qPCR in an independent set for randomly selected circRNAs.

As emerging epigenetic molecules, circRNAs are widely defined as a subset of endogenous RNAs that regulate target genes by binding to miRNAs [23]. A single circRNA might have several binding sites for miRNAs and therefore bind to different miRNAs simultaneously, thus relieving the depression of their target genes [24]. As the “molecular sponge”, circRNAs play important roles in several important physiological and pathological processes, including lymphocyte differentiation, immune responses, inflammation and oncogenesis [25,26,27]. In addition, circRNAs can contribute to the posttranscriptional regulation of other pathways, including absorbing RBPs to regulate the translation or production of proteins [28].

Immune surveillance plays a critical role in preventing leukemia’s development and progression. Our study investigated the crosstalk between epigenetics and immune surveillance, providing the molecular insight into immune evasion during AML relapse. We have identified the unique expression profile of circRNAs in the relapsed AML cells. In contrast with control HSPCs (HC and CR group), we confirmed that mRNAs regulated by DE circRNAs in relapse blasts are primarily involved in immune-related pathways. As is consistent with previous reports [4,29], our results demonstrate that T cell co-stimulation is impaired by relapse blasts, which might abolish AML recognition from donor-derived T cells and outgrow into clinically evident relapse. Furthermore, recent analyses have elucidated that B cell’s differentiation and function in the tumor microenvironment are typically associated with clinical outcomes and responses to immunotherapy [30]. Similarly, our results demonstrate altered expression profiles of the genes associated with B cell differentiation and the B cell receptor-signaling pathways (CD79B, BLNK, CD72, LYN, BTLA and IRF1) in the relapsed AML cells, indicating that B cells-mediated immune processes might have an effect on post-transplant relapse.

CircRNAs are demonstrated to function as miRNA sponges that regulate the downstream target genes. Several circRNAs have been linked to anti-tumor immune regulation through interactions with miRNAs [26,27,31]. Zhang et al. [32] demonstrated that hsa_circ_0020397 could upregulate the expression of PD-L1 by binding to miR-138 in colorectal cells, thus contributing to a tumor’s escape from immune responses. Other studies have also identified that circRNAs are connected to immune checkpoints and immunotherapy in many solid tumors [33,34]. However, the functions of circRNAs in the transplant immunity during leukemia are less known. In this study, a total of 10 circRNAs, 14 miRNAs and 16 hub mRNAs were identified in the ceRNA network, which they might target in patients with AML relapse, a process known to be heavily involved in the immune-evasion of leukemia cells. Two of these ten circRNAs have been reported to be associated with the pathogenesis and development of tumors. Hsa_circ_0012152 (circRNF220), which has been previously reported to have elevated expression in pediatric primary AML [17], was upregulated in the relapse blasts and works as a sponge of miR-92a-1-5p to upregulate the expression of PVR in our study. Another circRNA, hsa_circ_0002768 (circMYLK), was significantly upregulated and contributed to malignant development via targeting miR-34a to enhance the CCND3 expression in bladder cancer cells [35]. However, circMYLK was downregulated in the relapse blasts and inhibited the expression of HLA class II regulators (CIITA). These contrasting results demonstrate the various roles of the same circRNA in the different diseases may be associated with variations in downstream target miRNAs/mRNAs. Based on our bioinformatics analysis and RT-qPCR validation experiments, we first demonstrated that dysregulated circRNAs in the relapse samples modulate the expression of immune-related genes and are associated with allogeneic immune responses after transplant.

Notably, within downstream protein-coding genes controlled by DE circRNAs identified in the ceRNA network, we revealed some important transcripts that correlate with immune evasion. PVR (CD155), which is a novel immune checkpoint that binds to the T cell immunoreceptor with Ig and ITIM domains (TIGIT) to suppress immune responses, was highly expressed in the relapse blasts [4]. TNFSF4 (OX40L) is known as the cognate ligand for the tumor necrosis factor receptor OX40, which functions as a T cell co-stimulatory molecule [36]. Thus, the downregulation of TNFSF4 expression controlled by DE circRNAs has the potential to alleviate allogeneic T cell activity. In sum, deregulated circRNAs may exert their effects on immune escape through regulating immune-related genes, therefore contributing to leukemia relapse after transplantation.

## 5. Conclusions

In summary, this is the first study to investigate the comprehensive expression profile of circRNAs during AML relapse after allo-HSCT. Our data provides new insights into the molecular mechanisms associated with immune evasion during leukemia relapse after allo-HSCT, and therefore circRNAs may represent potential biomarkers for post-transplant relapse.

## 6. Limitations

This study has some limitations. First, the retrospective nature of the study, along with the small sizes of the BM samples, especially for diagnosis samples, have limited a more powerful conclusion. Furthermore, as our results were mainly obtained from bioinformatics models, further experimental studies are crucial to explore the exact role of these circRNAs in AML relapse. Despite the lack of the transcriptome information of diagnosis blasts, our findings provide clues for the roles of circRNAs in the AML development and relapse during transplantation. A larger scale analysis is warranted for more conclusive results.

## Figures and Tables

**Table 1:** Clinical Characteristics of the Individuals Enrolled in the Study.

	Discovery Set	Validation Set
No. of patients *	10	9
**Sex (F/M)**	6/4	4/5
**Age (Median, Range)**	42 (24~59)	40 (21~53)
**Risk Stratification (Standard Risk/High Risk)**	2/8	2/7
**MRD before HSCT (Negative/Positive)**	7/3	6/3
**Transplant Type (HID/MSD/MUD)**	5/5/0	4/5/0
**Time from HSCT to Sample Collection (d) (Median, IQR)**	156 (121~282)	147 (101~271)

* The basic information of healthy controls (HCs) is not presented in Table 1. High Risk: late European Group for Blood and Marrow Transplantation risk score; standard risk: early and intermediate European Group for Blood and Marrow Transplantation risk score; HSCT: hematopoietic stem cell transplantation; MRD before HSCT: Minimal residual disease (MRD) before transplantation, MRD was assessed in all patients by flow cytometry, karyotyping, WT1 and quantitative PCR in patients with abnormal karyotype; HID: haplo-identical donor; MSD: matched sibling donor; MUD: matched unrelated donor. IQR: interquartile range.

**Table 2:** The top 20 hub genes identified by cytoHubba.

Gene Symbol	MCC	log_2_FC(RE/CR)	*p* Value	log_2_FC(RE/HC)	*p* Value
CXCR4	69	−2.26	<0.001	−1.56	0.006
CD247	68	−3.51	<0.001	−2.42	<0.001
CXCL12	67	−5.44	<0.001	−4.73	<0.001
IL6	56	−4.01	0.002	−2.99	0.040
CD70	56	2.42	<0.001	2.33	<0.001
CD27	54	−2.68	<0.001	−1.20	0.024
CIITA	51	−2.64	0.019	−2.17	0.017
PVR	50	2.57	<0.001	2.06	0.030
LAT2	49	−1.12	0.047	−1.86	0.021
ITGAV	39	1.10	<0.001	1.36	<0.001
IL3RA	38	2.37	<0.001	2.08	0.003
IRF8	36	−2.98	<0.001	−3.21	<0.001
CD3G	35	−4.49	<0.001	−2.55	0.001
IL7R	35	−4.15	<0.001	−3.78	<0.001
TNFSF4	32	−2.86	<0.001	−2.50	0.040
IFNG	31	−4.21	0.001	−2.27	0.040
IL15	30	−2.91	0.003	−2.60	0.041
IRF4	28	−2.80	<0.001	−2.07	<0.001
CD79B	26	−3.08	<0.001	−2.37	<0.001
ITK	25	−5.06	<0.001	−3.42	<0.001

## Data Availability

The raw data supporting the conclusion of the present article will be made available by the author. Further inquiries can be directed to the corresponding author.

## Supplementary Materials

The following supporting information can be downloaded at: https://www.mdpi.com/article/10.3390/genes13111986/s1, Table S1. The primer sequences included in this study. Figure S1. The high viability and purity of CD34^+^ cells from the BM samples in the AML patients and healthy controls. Figure S2. Principal component analysis of circRNA expression profiles in AML patients with relapse (RE), remission (CR) and healthy controls (HC). Figure S3. CircRNAs expression profile in AML patients with remission (CR) and healthy controls (HC). Figure S4. DE circRNAs and mRNAs co-expression networks present in the CD34+ cells from patients with AML relapse versus remission. Figure S5. DE circRNAs and mRNAs co-expression networks present in the CD34+ cells from patients with AML relapse versus healthy controls.
