# Supplementary material for: Deregulated Expression of Circular RNAs Is Associated with Immune Evasion and Leukemia Relapse after Allogeneic Hematopoietic Stem Cell Transplantation
Source: Genes (Basel). 2022 Oct 31;13(11):1986. doi: 10.3390/genes13111986 (PMC9689715; doi:10.3390/genes13111986)
Supplement: Supplementary file 1 [file genes-13-01986-s001.zip › genes-1944841-supplementary.pdf]

Supplementary

Table S1. The primer sequences included in this study.

Gene		Primer sequences (5′-3′)
hsa_circ_0002768	Forward	GCCTTGTGATTCATGCTGTCC
	Reverse	CACATCCCCCATGGTCTTCT
hsa_circ_0003764	Forward	TCAAAATGAATCTCGTAGGCTGT
	Reverse	GTGCAAACGTTATGGGGTCT
hsa_circ_0012152	Forward	TGCTGTCTCTGGCCTCATTTC
	Reverse	GGGAATCATTCCCTCCTAAGAC
hsa_circ_0003828	Forward	GCACTATTGAGGAAACATCCAG
	Reverse	CCATCATAAGACCACTGCTG
hsa_circ_0008196	Forward	AGAGTGTAATGCTGTCCGCC
	Reverse	GCCTGGGATGCAGTATAGAGG
hsa_circ_0074775	Forward	AGGAGCTGCTGGCTGACATT
	Reverse	CTTCACCTCGCTGCAGAACC
GAPDH	Forward	GGGAAACTGTGGCGTGAT
	Reverse	AAGGGGTCATTGATGGCAAC
U6	Forward	TTAGCATGGCCCCTGC
	Reverse	TGCGTGTCGTGGAGTC
miR-211-3p	Forward	GTCGTATCCAGTGCGTGTCGTGG
	Reverse	AGTCGGCAATTGCACTGGATACG
miR-588	Forward	GCGTTGGCCACAATGGGT
	Reverse	AGTGCAGGGTCCGAGGTATT
miR-214-3p	Forward	TCGCCACAGCAGGCACAGACA
	Reverse	CTCAACTGGTGTCGTGGAGTCGGC
miR-152-5p	Forward	CAGAGGTTCTGTGATACACTC
	Reverse	GGTCCAGTTTTTTTTTTTTTTTAGTC
PVR	Forward	TGGAGGTGACGCATGTGTC
	Reverse	GTTTGGACTCCGAATAGCTGG
IL2RA	Forward	GTGGGGACTGCTCACGTTC
	Reverse	CCCGCTTTTTATTCTGCGGAA
CD247	Forward	GGCACAGTTGCCGATTACAGA
	Reverse	CTGCTGAACTTCACTCTCAGG
IFNG	Forward	TCGGTAACTGACTTGAATGTCCA
	Reverse	TCGCTTCCCTGTTTTAGCTGC

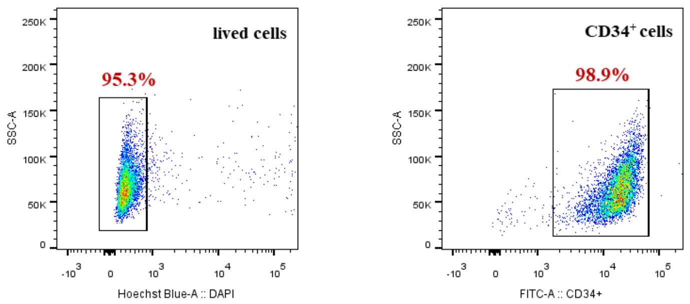

Figure S1. The high viability and purity of CD34⁺ cells from BM samples in AML patients and healthy controls.

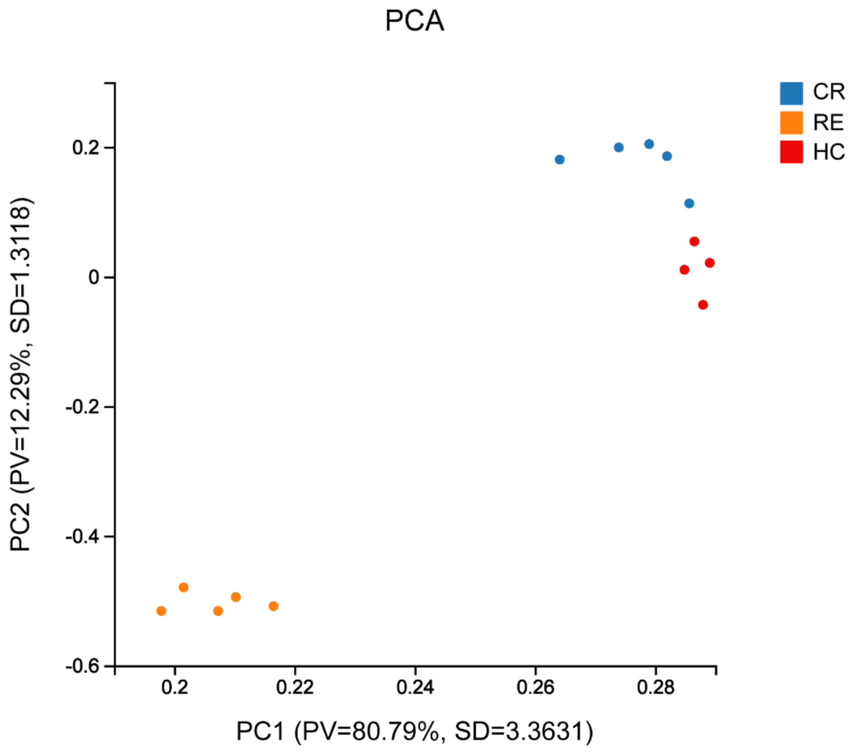

Figure S2. Principal component analysis of circRNA expression profiles in AML patients with relapse (RE), remission (CR) and healthy controls (HC).

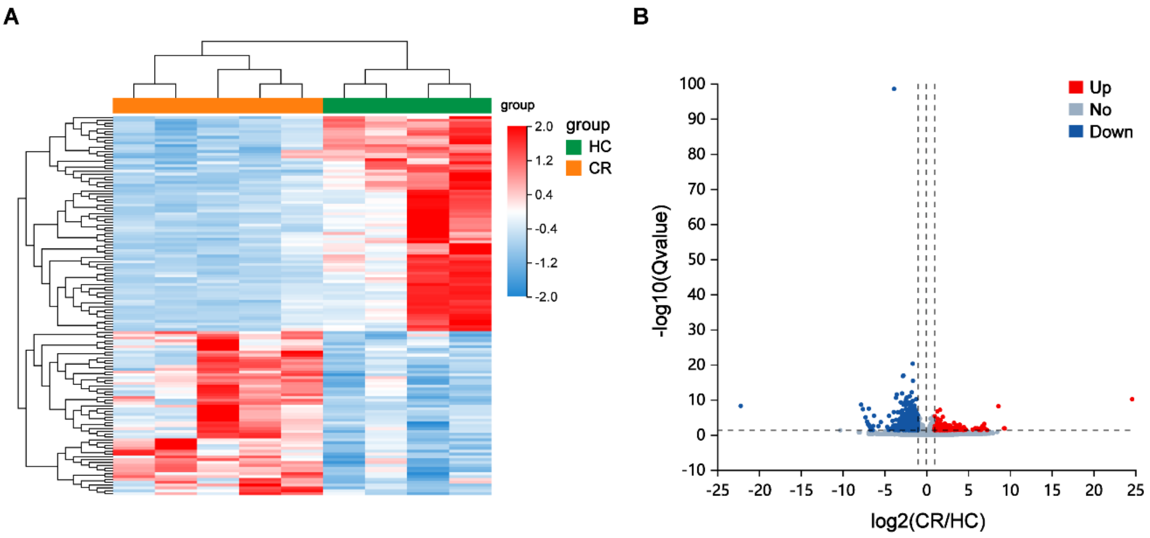

Figure S3. CircRNAs expression profile in AML patients with remission (CR) and healthy controls (HC). Heatmap **(A).** and volcano plot **(B)**. show that DE circRNAs expression profile between AML patients with remission (CR) and healthy controls (HC). Red indicates upregulated expression and blue represents downregulated expression.

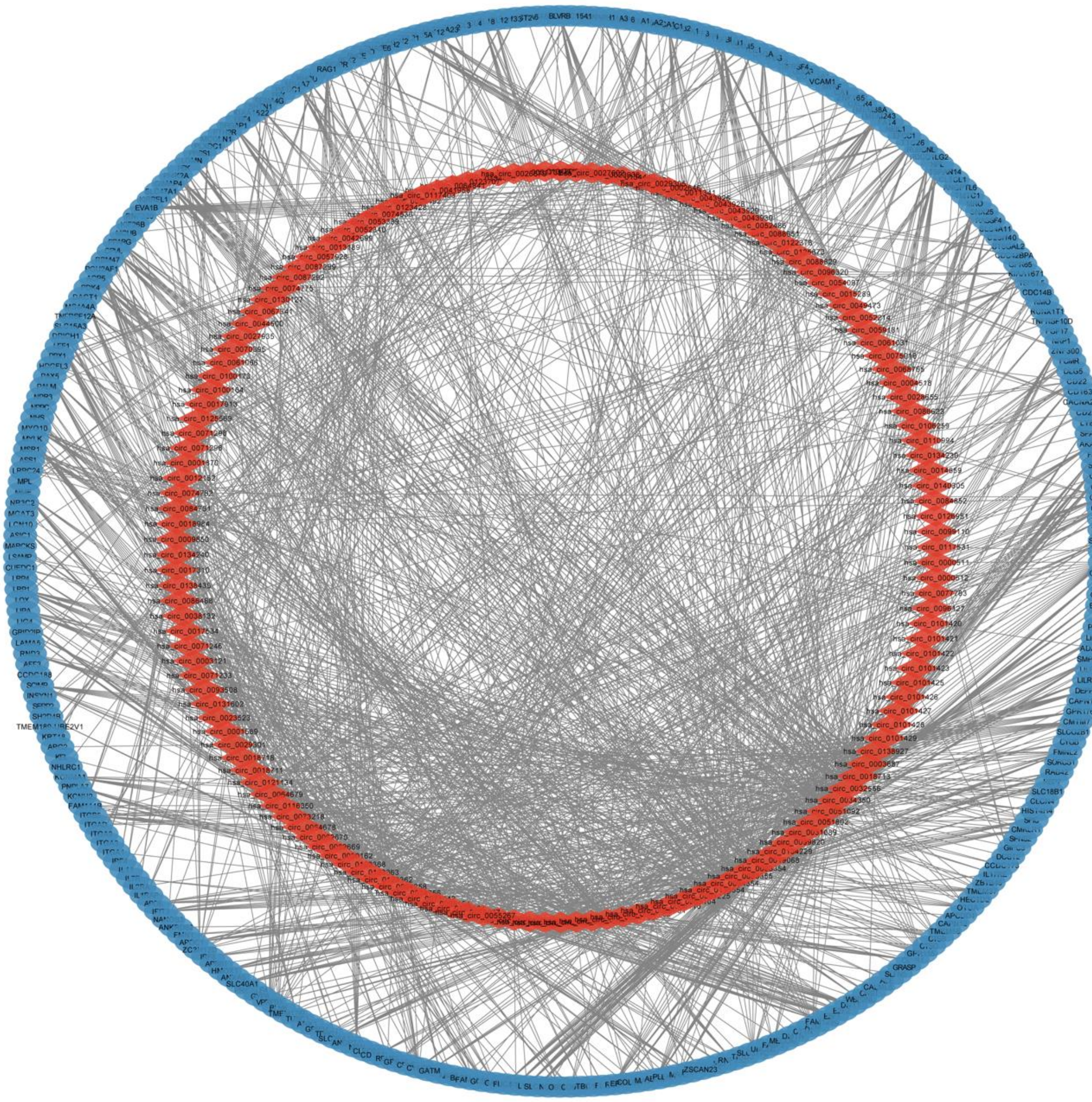

Figure S4. DE circRNAs and mRNAs co-expression network present in the CD34+ cells from patients with AML relapse versus remission. The red square nodes represent circRNAs, and the blue circular nodes denote mRNAs.

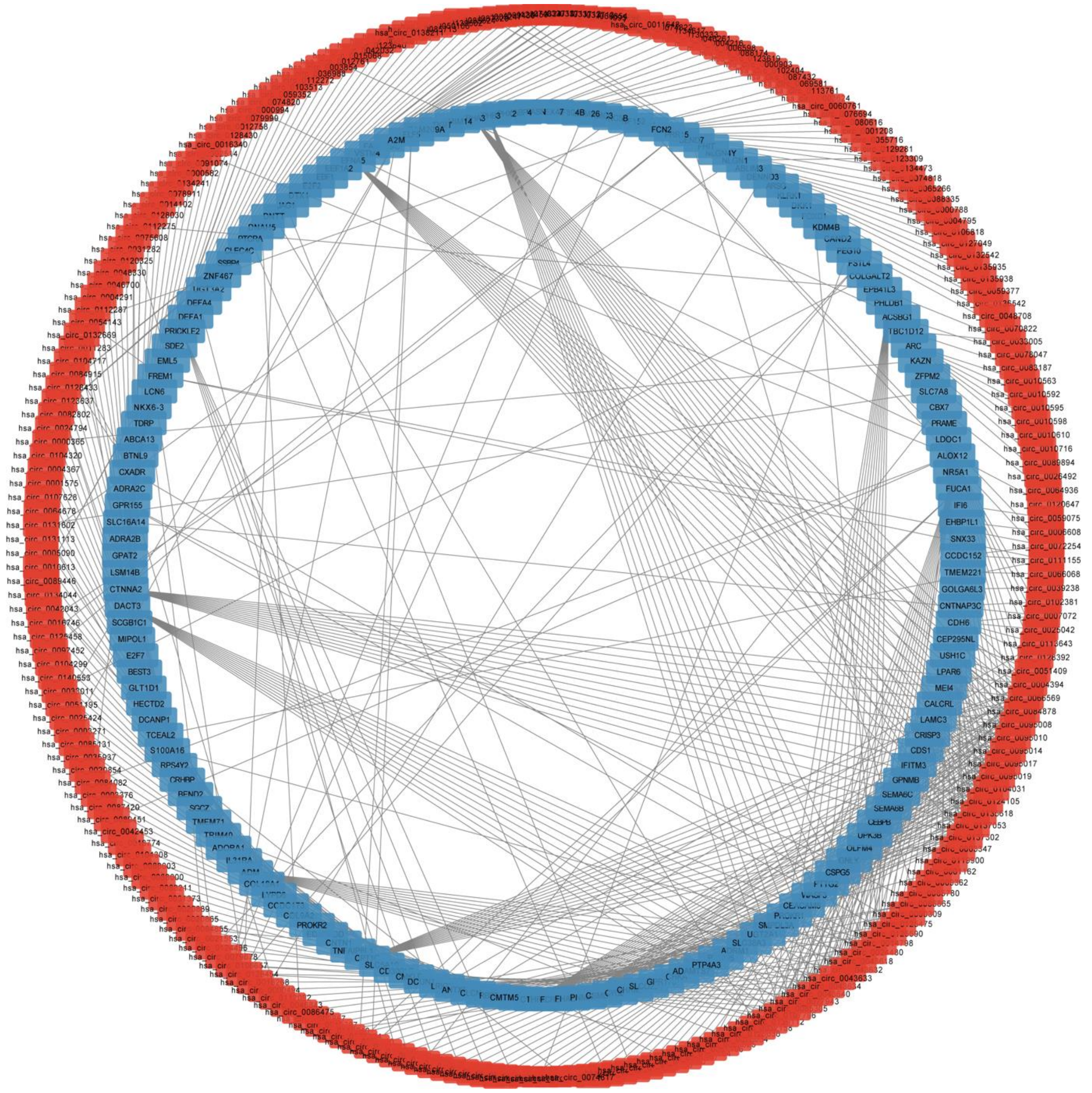

Figure S5. DE circRNAs and mRNAs co-expression network present in the CD34+ cells from patients with AML relapse versus healthy controls. The red square nodes represent circRNAs, and the blue circular nodes denote mRNAs.
